# DNA methylation at the *Igf2/H19* imprinting control region is associated with cerebellum mass in outbred mice

**DOI:** 10.1186/1756-6606-5-42

**Published:** 2012-12-06

**Authors:** Ruth Pidsley, Cathy Fernandes, Joana Viana, Jose L Paya-Cano, Lin Liu, Rebecca G Smith, Leonard C Schalkwyk, Jonathan Mill

**Affiliations:** 1 Institute of Psychiatry, King’s College London, De Crespigny Park, Denmark Hill, London, SE5 8AF, UK; 2 University of Exeter Medical School, University of Exeter, Magdalen Road, Exeter, EX1 2LU, UK

**Keywords:** *Igf2*, *H19*, Epigenetics, DNA methylation, Cerebellum, Brain, Mouse, Genotype, Genomic imprinting

## Abstract

**Background:**

*Insulin-like growth factor 2 (Igf2)* is a paternally expressed imprinted gene regulating fetal growth, playing an integral role in the development of many tissues including the brain. The parent-of-origin specific expression of *Igf2* is largely controlled by allele-specific DNA methylation at CTCF-binding sites in the imprinting control region (ICR), located immediately upstream of the neighboring *H19* gene. Previously we reported evidence of a negative correlation between DNA methylation in this region and cerebellum weight in humans.

**Results:**

We quantified cerebellar DNA methylation across all four CTCF binding sites spanning the murine *Igf2/H19* ICR in an outbred population of Heterogeneous Stock (HS) mice (n = 48). We observe that DNA methylation at the second and third CTCF binding sites in the *Igf2/H19* ICR shows a negative relationship with cerebellar mass, reflecting the association observed in human post-mortem cerebellum tissue.

**Conclusions:**

Given the important role of the cerebellum in motor control and cognition, and the link between structural cerebellar abnormalities and neuropsychiatric phenotypes, the identification of epigenetic factors associated with cerebellum growth and development may provide important insights about the etiology of psychiatric disorders.

## Background

Genomic imprinting regulates the monoallelic expression of genes in a parent-of-origin specific manner. To date >90 imprinted loci have been identified in the mouse genome [1], many residing in co-regulated clusters with other imprinted genes, and their allele-specific expression controls a diverse range of functions including growth and development [1]. Of note, the imprinted *insulin-like growth factor 2 (Igf2)* gene located on mouse distal chromosome 7, along with other genes in the insulin and insulin-like growth factor regulatory pathway, has been shown to be integral for fetal growth and the development of many tissues including the brain [2-5]*.*

**Figure 1:**
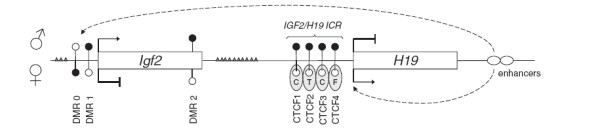
**Schematic map of the mouse *Igf2/H19 *locus, with paternal allele (♂) on the top and maternal allele (♀) on the bottom.** DMRs are represented by circles: filled circles indicate a typically methylated allele and empty circles a typically unmethylated allele. In the current study, DNA methylation was assessed across 5 amplicons spanning the 1st-4th CTCF binding sites of the *Igf2/H19* ICR (CTCF1, CTCF2, CTCF3 and CTCF4). SNPs genotyped are shown as gray triangles (from left to right rs33816033, rs33820807, rs51306385, rs33820056, rs33815374, rs33818240, rs33816897, rs33816896, rs33815163, rs33818772, rs33817683, rs33816812, rs33818680).

In most somatic cells *Igf2* and the neighboring *H19* gene are reciprocally imprinted; *Igf2* gene expression is silenced on the maternal allele, whereas *H19* is silenced on the paternal allele. In mice this allele-specific expression is associated with allele-specific DNA methylation at several differentially methylated regions (DMRs) in the *Igf2* gene and at the *Igf2/H19* imprinting control region (ICR) located immediately upstream of the *H19* promoter [6]. The ICR contains four methylation-sensitive CCCTC-binding factor (CTCF) binding-sites mediating the assembly of a chromatin insulator that blocks interactions between the *Igf2* promoter and enhancers downstream of the *H19* gene. On the unmethylated maternal allele, CTCF binds to the ICR silencing *Igf2* expression and stimulating the transcription of *H19* (Figure 1). IGF2 is a major driver of prenatal growth – for example, placenta specific *Igf2* transcripts control the growth of the placenta and supply of maternal nutrients to the developing fetus [7]. IGF2 also acts as part of the IGF signaling pathway to regulate postnatal growth of somatic tissues including the brain [5,8]. The *H19* gene encodes an untranslated RNA which acts as a trans-regulator of the imprinted gene network controlling embryonic growth in mice [9]. 

Despite being a classically imprinted region, with clear-cut patterns of allele-specific gene expression, there is evidence of considerable epigenetic heterogeneity at the *Igf2/H19* locus. Population studies have shown that DNA methylation across the homologous region in the human genome is a normally distributed quantitative trait, which can be influenced by stochastic, genetic and environmental factors [10,11]. For example, individuals conceived during famine show alterations in *IGF2* methylation [12], whilst polymorphisms in the region show an independent and additive association with DNA methylation [10]. Even isogenic strains of mice raised in a controlled environment demonstrate evidence of epigenetic heterogeneity; allelic DNA methylation levels at the *Igf2/H19* locus vary extensively in a tissue-specific manner during fetal development, primarily on the expressed paternal allele [13].

The cerebellum is a region of the brain with an important role in motor control and cognition [14]. The cerebellum has a protracted period of development [15] and ultimately accounts for ~12% of total brain weight [16]. Only a handful of studies have investigated *Igf2* expression and its regulation by genomic imprinting in the mouse cerebellum. Early work revealed that the *Igf2* gene is paternally expressed in cerebellar granule and glial cells during early postnatal development [17], with expression being spatially and temporally specific, indicating that the local synthesis of IGF2 is important for cerebellar development. A recent study showed that *Igf2* regulates the proliferation of granule cell precursors, which ultimately determine the final size and shape of the cerebellum [18]. Together these results indicate that the regulation of *Igf2* expression during early postnatal development likely plays an important role in the development of the cerebellum.

Previously we reported evidence of a strong, negative correlation between DNA methylation at the *IGF2/H19* ICR and cerebellum weight in humans [11]. However, we observed no relationship between *IGF2/H19* DNA methylation in the frontal cortex and net brain mass, suggesting that the association is cerebellum-specific. In the current study we comprehensively quantify cerebellar DNA methylation at all four CTCF binding sites spanning the murine *Igf2/H19* ICR in an outbred population of Heterogeneous Stock (HS) mice. We report that epigenetic variation in the region shows an association with between-individual differences in cerebellar mass, reflecting the association observed in human post-mortem brain tissue.

## Results

### Structure of cerebellar DNA methylation across the murine ICR

**Figure 2:**
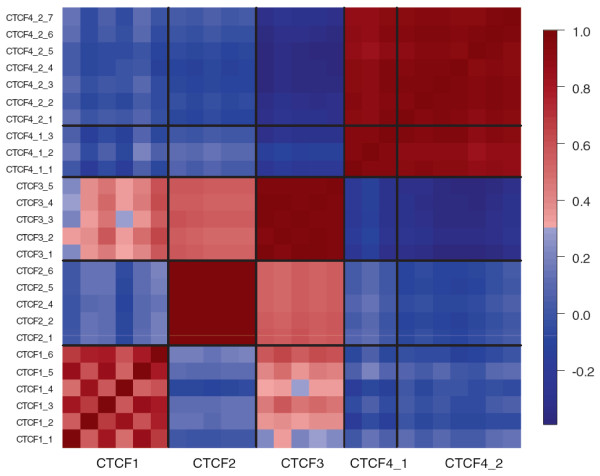
**Correlation between DNA methylation at individual CpG sites within the mouse *Igf2/H19 *ICR, assayed using five amplicons spanning the four CTCF binding sites.** CpG sites for each amplicon are numbered according to the CpG sites in Additional file 1: Materials 1. Two CpG sites have been removed after quality control steps, as described in Additional file 1: Table S2.

Figure 2 shows the correlation between DNA methylation at all 26 CpG sites spanning the four CTCF binding sites assessed in this study, using 5 amplicons: CTCF1, CTCF2, CTCF3, CTCF4_1 and CTCF4_2. Because DNA methylation is strongly positively correlated between individual CpG sites within each amplicon, the amplicon-mean DNA methylation level was used for all subsequent analyses. DNA methylation was highly correlated between the two adjacent amplicons covering CTCF4 (CTCF4_1 and CTCF4_2). Interestingly, although DNA methylation is largely positively correlated across the amplicons spanning CTCF1, 2 and 3, it is negatively correlated between these amplicons and those spanning CTCF4.

### Association of *Igf2/H19* DNA methylation with cerebellum mass

**Figure 3:**
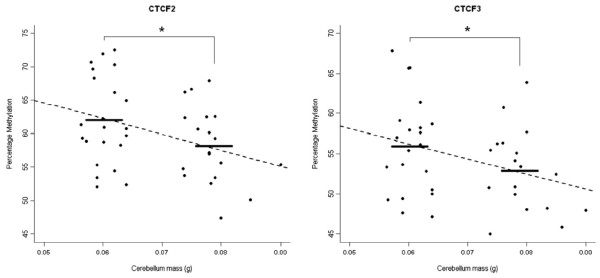
**Epigenetic variation at the mouse *Igf2/H19 *ICR is associated with cerebellum mass.** DNA methylation in the cerebellum across amplicons spanning CTCF2 and CTCF3 is significantly different between mice with high and low cerebellar mass (CTCF2: t(39) = −1.97, p = 0.028; CTCF3: t(37) = −1.72, p = 0.047). Furthermore, cerebellar DNA methylation at CTCF2 and CTCF3 shows a linear relationship with cerebellum mass (CTCF2: β = −0.35, p = 0.023; CTCF3: β = −0.33, p = 0.041).

Mean DNA methylation across amplicons CTCF2 and CTCF3 was significantly lower in the high cerebellar mass group than the low cerebellar mass group (CTCF2: t(39) = −1.97, mean high cerebellum mass =  58.1%, mean low cerebellum mass = 61.9%, p = 0.028; CTCF3: t(37) = −1.72, mean high cerebellum mass = 52.9%, mean low cerebellum mass = 55.9%, p = 0.047) (Figure 3). Furthermore, cerebellar DNA methylation at CTCF2 and CTCF3 showed a significant negative relationship with cerebellum mass at both amplicons (CTCF2: β = −0.35, t(39) = −2.37, p-value = 0.023; CTCF3: β = −0.33, t(37) = −2.12, p-value = 0.041) (Figure 3). No significant relationship was observed between DNA methylation at any other CTCF region and cerebellum mass. Of note, no association was observed between cerebellar DNA methylation and net brain mass at either amplicon, suggesting that the findings we observe are cerebellum-specific. Finally, we tested whether this effect is specific to DMRs regulating the monoallelic expression of *Igf2* and *H19* by quantifying DNA methylation across DMRs associated with four additional brain-expressed imprinted genes (*Kcnq1ot1, Mcts2, Nap1l5,* and *Sgce*). No between-group differences in DNA methylation were observed between animals with high and low cerebellar mass (Additional file 1: Figure S1).

Multiple linear regression was used to further investigate the relationship between cerebellar mass and DNA methylation at CTCF2 and CTCF3, including animal batch and net brain mass as covariates in the analysis. For CTCF2 the overall regression model was significant (adjusted R-squared = 0.184, F = 4.00 p = 0.015) and explained approximately 18% of the variance in cerebellum mass, with DNA methylation (β = −0.31, p = 0.041) and net brain mass being significantly predictive (β = 0.38, p = 0.031). The regression model for CTCF3 was also significant (adjusted R-squared = 0.224, F = 4.65, p = 0.008), explaining approximately 22% of the variance in cerebellum mass, with DNA methylation (β = −0.36, p = 0.023) and net brain mass both being predictive (β = 0.48, p = 0.006). Hierarchical linear regression was employed to determine the unique contribution of net brain mass and DNA methylation to the variance observed in cerebellar mass. As expected, net brain weight was significantly associated with cerebellum weight, explaining 13% of the variance (R-squared = 0.130, F = 6.993, p = 0.012) for the samples included in the CTCF2 analysis and 14% of the variance (R-squared = 0.144, F = 7.374, p = 0.010) for the samples included in the CTCF3 analysis. DNA methylation, added in the second step of the analysis, was also a significant contributor to the observed variance in cerebellar mass, with CTCF2 methylation explaining 7% of the variance (ΔR-squared = 0.071, ΔF = −0.944, p = 0.005) and CTCF3 methylation explaining 8% of the variance (ΔR-squared = 0.075, ΔF =−1.056, p = 0.004) respectively.

### Genetic analyses

In addition to quantifying DNA methylation, we genotyped 13 polymorphic single nucleotide polymorphisms (SNPs) spanning the *Igf2* and *H19* imprinted gene region (chr7:149745781–149890507). Standard genetic association analyses showed that none of the SNPs tested were directly associated with total brain mass, cerebellum mass, or DNA methylation either individually or as multi-marker haplotypes.

## Discussion

These data provide further evidence that epigenetic variation at the *Igf2/H19* ICR is associated with growth and development of the cerebellum. To our knowledge this is the first detailed investigation of DNA methylation across all four CTCF binding sites in the *Igf2/H19* ICR in mouse cerebellum. Our results suggest that cerebellar DNA methylation across the 2nd and 3rd CTCF-binding sites, upstream of *H19*, shows a negative relationship with cerebellum mass, with ~7% of the variance in cerebellum mass being uniquely explained by DNA methylation at these loci. No significant relationship was observed between DNA methylation and overall net brain mass for either amplicon, indicating that the effect of cerebellar DNA methylation at these sites is likely to be cerebellum-specific. The results from this study concur with our previous data, which highlight a negative relationship between DNA methylation in the *IGF2/H19* ICR in post-mortem human cerebellum tissue and cerebellum mass measured at autopsy. Although the human association was primarily mediated by altered DNA methylation at the 3rd CTCF binding site, it is hard to directly compare specific locations across species because there is incomplete sequence homology between the mouse and human genome at this region – for example the murine ICR contains four CTCF binding sites whereas the human ICR contains seven.

Epigenetic heterogeneity in the *IGF2/H19* ICR is a normally distributed quantitative trait influenced by stochastic, genetic and environmental factors [19]. We observe considerably more inter-individual variation at this locus in human compared to mouse cerebellum, which may result from the fact that the HS mice used in the current study were maintained in a controlled laboratory housing environment and, although outbred, have limited genetic variation compared to humans. Indeed when we investigated cis-acting genetic factors we found no association with DNA methylation, suggesting that the reported DNA methylation differences are most likely to result from stochastic factors, trans-acting genetic factors, or unmeasured environmental factors. Of note, nutrition early in development is associated with decreased cerebellum mass and alterations to the IGF system within the cerebellum [20]. Although the HS mice in this study were given a standard controlled diet, they may have been exposed to subtle differences in their rearing – for example, maternal behavior and suckling prior to weaning – that could also have influenced DNA methylation.

The mouse cerebellum continues to grow for three weeks postnatally, at which point its circumference has reached adult size [21]. Corresponding expression data suggests that *Igf2* mRNA levels are coordinated with specific growth patterns in the brain [17]. In this study we have looked at DNA methylation in adult mice to serve as a proxy for levels of gene expression during development. Investigations into the factors determining cerebellar growth and development are important as a growing body of work from human and animal studies suggests that the role of the cerebellum may extend beyond the regulation and coordination of motor function, to attention, perception, working memory and spatial orientation [14]. In humans, cerebellar abnormalities are among the most consistently reported structural findings in autism and attention deficit hyperactivity disorder [22], and progressive loss of cerebellar volume has been reported in childhood-onset schizophrenia and other types of psychosis [23].

The current study has a number of limitations. First, the absolute mean DNA methylation difference between the high and low cerebellar groups is relatively modest (~4%), but is consistent across the multiple adjacent CpG sites assessed in the CTCF2 and CTCF3 amplicons. Previous work has shown that *Igf2* expression in the cerebellum is highly cell-type specific [17], and the small absolute difference we observe may result from much larger changes in a specific subpopulation of cells. Second, although the animals were maintained in a controlled environment, and we were careful to control for variables such as batch and net brain mass in our analyses, it is possible that our findings are biased by unmeasured variables or confounding influences such as maternal behavior. Third, the sodium bisulfite conversion method does not distinguish between methylated cytosines and hydroxymethylated cytosines, which may be particularly important here as hydroxymethylation is relatively enriched in the Purkinje cells of the cerebellum [24]. Fourth, we did not have access to RNA from the same samples, so we are unable to relate our epigenetic data to changes in steady-state mRNA levels. Our previous work [11] in humans, however, did not report an association between altered DNA methylation at this region and absolute levels of gene expression. It is possible that the observed DNA methylation changes result in allelic skewing of expression, rather than changes in absolute expression values or that DNA methylation may be a mark of gene expression changes occurring during development, rather than current gene expression levels. Fifth, we only analysed DNA methylation in the cerebellum itself, so we cannot assess the tissue-specificity of the findings. However, our previous work in humans looking at several brain regions [11] indicated that the association between *IGF2/H19* DNA methylation and brain region weight was cerebellum-specific. Further work would be required to assess the tissue-specificity of the finding in mouse. Finally, we only assessed cerebellum mass and DNA methylation at one time point in adulthood, so could not examine changes occurring during development.

To conclude, we show that epigenetic variation across the *Igf2/H19* ICR shows a significant negative relationship with cerebellar mass, with hypomethylation at specific CTCF binding motifs being associated with increased cerebellar mass, reflecting our previous work in human tissue. Future work will expand these analyses to look at brain-region and cell-type specific effects, to determine the genetic and environmental factors underlying the changes in *Igf2/H19* DNA methylation and to explore the relationship between DNA methylation and gene expression and measures of behavior.

## Methods

### Animals and brain tissue samples

All housing and experimental procedures were performed in compliance with the UK Home Office Animals Scientific Procedures Act 1986. Male HS mice were shipped from the Institute for Behavioral Genetics, University of Colorado at Boulder (Boulder, USA) to the UK at the age of approximately eight weeks. Mice were individually identified upon arrival, and were singly housed and habituated under strictly controlled housing condition at the Institute of Psychiatry, King’s College London. The animals used in this study had an average age of 134.4 days (+/− 11.6). Individual animals were weighed and killed by cervical dislocation. Immediately after cervical dislocation, the whole brain including olfactory bulbs and brainstem was carefully removed from the skull and placed on the ventral side (dorsal facing up) on a sterile petri dish containing ice-cold artificial cerebrospinal fluid over ice. The brains were transected at the caudal margin of the cerebellum and the olfactory bulbs dissected out by cutting the fissura rhinalis using a surgical blade. Wet cerebri (whole brain minus cerebellum and olfactory bulbs) were briefly rolled on filter paper to dry excess aCSF and weighed immediately to the nearest 0.1 mg (net brain mass). Similarly, the dissected cerebellums were rolled in filter paper and weighed (cerebellum mass). Tissue samples were snap frozen on dry ice and stored at −80°C until used for DNA extraction. Because samples came from three experimental batches of HS mice, cerebellar mass was scaled and centered separately by batch. The resulting distribution was approximately normal with no age effect, and using these standardized values 48 mice were selected from a total of 274 from three batches as representing extremes of cerebellar mass in order to increase the statistical power to detect an association between DNA methylation and phenotype [25]. The descriptive statistics for these animals are given in Table 1. 

**Table 1:** Uncorrected wet brain and cerebellum mass (mean ± SD) for the HS mice used in this study, split by a) batch and b) high and low cerebellum mass

**a)**	**n**	**Age (days)**		**Cerebellum mass (g)**		**Net brain mass (g)**	
Batch A	13	136.6	± 3.5	0.072	± 0.011	0.39	± 0.03
Batch B	17	120.1	± 4.1	0.071	± 0.010	0.39	± 0.02
Batch C	18	145.9	± 1.1	0.067	± 0.009	0.35	± 0.02
**Total**	**48**	**134.3**	± **11.6**	**0.070**	± **0.010**	**0.37**	± **0.03**
**b)**							
High	24	133.6	± 12.2	0.079	± 0.004	0.38	± 0.02
Low	24	134.9	± 11.2	0.060	± 0.003	0.36	± 0.02
**Total**	**48**	**134.3**	± **11.6**	**0.070**	± **0.010**	**0.37**	± **0.03**

### DNA methylation analysis

DNA was extracted from the cerebellar samples using the Qiagen AllPrep DNA/RNA Mini Kit (Qiagen, Valencia, CA, USA) according to the manufacturer’s standard protocol. Sodium bisulfite conversion of 500 ng genomic DNA was performed in duplicate using the EZ-96 DNA Methylation kit (Zymo Research, CA, USA), following the manufacturer's standard protocol. Bisulfite pyrosequencing was used to quantify DNA methylation at individual CpG sites within the *Igf2* ICR. Primer sequences for 4 amplicons spanning CTCF binding sites 2, 3 and 4 were acquired from a previously published study [26]. Primers for CTCF binding site 1 were designed using the PyroMark Assay Design software 2.0 (Qiagen, UK). Amplicons are referred to as CTCF1, CTCF2, CTCF3, CTCF4_1 and CTCF4_2, and their locations can be seen in Figure 1. Primer sequences for each amplicon are given in Table 2 and the genomic sequences of each amplicon are given in the Additional file 1: Materials 1. Bisulfite-PCR amplification was performed in duplicate using optimized cycling conditions (see Table 2). DNA methylation was quantified using the Pyromark Q24 system (Qiagen, UK) following the manufacturer’s standard instructions and the Pyro Q24 CpG 2.0.6 software. DNA methylation across DMRs associated with four additional imprinted genes (*Kcnq1ot1, Mcts2, Nap1l5,* and *Sgce*) was assessed using Sequenom EpiTYPER as described previously [27]. Assay details are given in Additional file 1: Table S1. 

**Table 2:** Details of the bisulfite-pyrosequencing DNA methylation assays utilized in this study

**Regions**	**Position**	**PCR primers (5′ to 3′)**	**Annealing temperature (°C)**	**Cycle number**	**Product length (bp)**	**Sequencing primers (5′ to 3′)**	**CpGs**	**Coordinates in NCBI37/mm9**
CTCF binding site 1	F	TTGTTGAATTAGTTGTGGGGTTTA	56	46	149	GAATTAGTTGTGGGGTTTATA	6	chr7:149,767,865-149,768,017
	R	Biotin-ATTCCAATACCAAAAATAAAAAAACTCT						
CTCF binding site 2	F	Biotin-AAAGAATTTTTTGTGTGTAAAGATT	56	46	168	AACTCAATCAATTACAATCC	6	chr7:149,767,573-149,767,748
	R	ATCAAAAACTAACATAAACCCCTAAC						
CTCF binding site 3	F	GGGTTTTTTTGGTTATTGAATTTTAA	56	46	224	TGTTATGTGTAATAAGGGAA	6	chr7:149,766,533-149,766,776
	R	Biotin-AATACACACATCTTACCACCCCTATA						
CTCF binding site 4	F	Biotin-TTTTTGGGTAGTTTTTTTAGTTTTG	56	46	211	CTATAACCAAATCTACACAA	5	chr7:149,766,109-149,766,322
	R	ACACAAATACCTAATCCCTTTATTAAAC				ACTCAAAACTTTATCACAAC	7	

Primers for CTCF binding site 1 were designed using PyroMark Assay Design software 2.0 (Qiagen, UK). Primer sequences for all other CTCF binding sites were obtained from a previously published study *(Fauque P et al. Hum Mol Genet.*[2010]*).*

### Statistical analysis

Prior to analysis, stringent data quality control was performed to remove potentially unreliable measurements of DNA methylation (see Additional file 1: Table S2). Non-CpG cytosines were used as internal controls to verify the efficiency of bisulfite conversion. No indication of incomplete bisulfite conversion was observed for any sample. All statistical analysis was performed within the “R” statistical programming environment ( http://www.R-project.org). Spearman’s rank correlation tests were used to test whether DNA methylation was coordinated within and between the CTCF binding sites. As CpG sites within each of the amplicons were strongly and significantly correlated (see Figure 2), mean DNA methylation scores for the five amplicons were used in all subsequent analyses. One-sided t-tests were used to test our hypothesis that high cerebellar mass is associated with relative hypomethylation at the *Igf2/H19* ICR CTCF binding sites. Linear regression was subsequently used to explore any observed association between cerebellar mass and DNA methylation. A simple bias correction method was used to correct for only profiling DNA methylation in mice with extremes of cerebellar mass [25]. Multiple linear regression was used to examine other predictors of cerebellum mass; additional explanatory variables in the model were batch and net brain mass. Hierarchical linear regression models were used to assess the unique contribution of DNA methylation in these models. To investigate the specificity of our analyses to the cerebellum we also used linear regression to test for an association between net brain mass and cerebellar DNA methylation.

### Genotype analysis

13 polymorphic SNPs spanning the *Igf2/H19* imprinted region (chr7:149745781–149890507) were selected for genotype analysis. The SNPs were extracted from existing microarray data obtained from 40 of the mice included in this study (20 high and 20 low cerebellum mass). Samples were genotyped using the Affymetrix Mouse Diversity Genotyping Array and analysis performed using the MouseDivGeno package in R ( http://cgd.jax.org/tools/mousedivgeno) [28]. Haploview 4.2 ( http://www.broadinstitute.org/haploview) was used to establish that all 13 SNPs were in Hardy-Weinberg Equilibrium (HWE). Lewontin’s D’ and the linkage disequilibrium (LD) coefficient r^2^ were calculated using Haploview to measure the LD between all pairs of biallelic loci. HWE p-values, minor allele frequencies and LD between the SNPs are given in Additional file 1: Table S3 and Additional file 1: Figure S2. ANOVA was used to test each SNP for an association with total brain mass, cerebellum mass, and mean percentage cerebellar DNA methylation at each of the five amplicons. To further interrogate the association between genotype and DNA methylation we used UNPHASED 3.1.4 ( http://www.mrc-bsu.cam.ac.uk/personal/frank/software/unphased) to test the association between haplotypes and mean cerebellum DNA methylation values.

## Abbreviations

CTCF: CCCTC-binding factor; Igf2: Insulin-like growth factor 2; LD: Linkage disequilibrium; DNA: deoxyribonucleic acid; RNA: ribonucleic acid; ICR: imprinting control region; HS: Heterogeneous Stock; PCR: polymerase chain reaction; DMR: differentially methylated region; SNP: single nucleotide polymorphism.

## Competing interests

The authors' declare that they have no competing interests.

## Authors’ contributions

RP did the lab work and statistical analyses. RP and JM conceived the study and drafted the manuscript. LS, CF, JP-C and LL reared the animals, dissected the brains, and measured brain/cerebellum mass. LS selected mouse samples for analysis. JV and RS helped with the revisions to the manuscript. All authors read and approved the final manuscript.

## Supplementary Material

Additional file 1 **Supplementary Materials 1.** Genomic sequences of each amplicon used in this study. **Figure S1.** No differences in DNA methylation at four additional imprinted loci (*Kcnq1ot1, Mcts2, Nap1l5* and *Sgce*). **Figure S2.** High linkage disequilibrium (LD) across the SNPs genotyped in the murine *Igf2/H19* region. **Table S1.** Details for the Sequenom EpiTYPER assays used to profile DNA methylation across DMRs associated with *Kcnq1ot1, Mcts2, Nap1l5* and *Sgce.***Table S2.** Stringent quality control and filtering steps used in analysis of *Igf2/H19* DNA methylation data. **Table S3.** LD between the SNPs genotyped in *Igf2* and *H19.* Click here for file

## References

[B1] Morison IM Ramsay JP Spencer HG A census of mammalian imprinting Trends Genet 2005 21 8 457 465 10.1016/j.tig.2005.06.008 15990197

[B2] Reik W Walter J Genomic imprinting: parental influence on the genome Nat Rev 2001 2 1 21 32 10.1038/35047554 11253064

[B3] Kent LN Ohboshi S Soares MJ Akt1 and insulin-like growth factor 2 (Igf2) regulate placentation and fetal/postnatal development Int J Dev Biol 2012 56 4 255 261 10.1387/ijdb.113407lk 22562201 PMC3894249

[B4] D'Ercole AJ Ye P Calikoglu AS Gutierrez-Ospina G The role of the insulin-like growth factors in the central nervous system Mol Neurobiol 1996 13 3 227 255 10.1007/BF02740625 8989772

[B5] Bracko O Singer T Aigner S Knobloch M Winner B Ray J Clemenson GD Jr Suh H Couillard-Despres S Aigner L Gene expression profiling of neural stem cells and their neuronal progeny reveals IGF2 as a regulator of adult hippocampal neurogenesis J Neurosci 2012 32 10 3376 3387 10.1523/JNEUROSCI.4248-11.2012 22399759 PMC3338187

[B6] Phillips JE Corces VG CTCF: master weaver of the genome Cell 2009 137 7 1194 1211 10.1016/j.cell.2009.06.001 19563753 PMC3040116

[B7] Constancia M Hemberger M Hughes J Dean W Ferguson-Smith A Fundele R Stewart F Kelsey G Fowden A Sibley C Placental-specific IGF-II is a major modulator of placental and fetal growth Nature 2002 417 6892 945 948 10.1038/nature00819 12087403

[B8] Lui JC Finkielstain GP Barnes KM Baron J An imprinted gene network that controls mammalian somatic growth is down-regulated during postnatal growth deceleration in multiple organs Am J Physiol Regul Integr Comp Physiol 2008 295 1 R189 196 10.1152/ajpregu.00182.2008 18448610 PMC2494817

[B9] Gabory A Ripoche MA Le Digarcher A Watrin F Ziyyat A Forne T Jammes H Ainscough JF Surani MA Journot L H19 acts as a trans regulator of the imprinted gene network controlling growth in mice Development 2009 136 20 3413 3421 10.1242/dev.036061 19762426

[B10] Tobi EW Slagboom PE van Dongen J Kremer D Stein AD Putter H Heijmans BT Lumey LH Prenatal Famine and Genetic Variation Are Independently and Additively Associated with DNA Methylation at Regulatory Loci within IGF2/H19 PLoS One 2012 7 5 e37933 10.1371/journal.pone.0037933 22666415 PMC3364289

[B11] Pidsley R Dempster E Troakes C Al-Sarraj S Mill J Epigenetic and genetic variation at the IGF2/H19 imprinting control region on 11p15.5 is associated with cerebellum weight Epigenetics 2011 7 2 155 163 22395465 10.4161/epi.7.2.18910 PMC3335909

[B12] Heijmans BT Tobi EW Stein AD Putter H Blauw GJ Susser ES Slagboom PE Lumey LH Persistent epigenetic differences associated with prenatal exposure to famine in humans Proc Natl Acad Sci U S A 2008 105 44 17046 17049 10.1073/pnas.0806560105 18955703 PMC2579375

[B13] Weber M Milligan L Delalbre A Antoine E Brunel C Cathala G Forne T Extensive tissue-specific variation of allelic methylation in the Igf2 gene during mouse fetal development: relation to expression and imprinting Mech Dev 2001 101 1–2 133 141 11231066 10.1016/s0925-4773(00)00573-6

[B14] Martin LA Goldowitz D Mittleman G The cerebellum and spatial ability: dissection of motor and cognitive components with a mouse model system Eur J Neurosci 2003 18 7 2002 2010 10.1046/j.1460-9568.2003.02921.x 14622233

[B15] Haddara MA Nooreddin MA A quantitative study on the postnatal development of the cerebellar vermis of mouse J Comp Neurol 1966 128 2 245 254 10.1002/cne.901280209 4165738

[B16] Airey DC Lu L Williams RW Genetic control of the mouse cerebellum: identification of quantitative trait loci modulating size and architecture J Neurosci 2001 21 14 5099 5109 11438585 10.1523/JNEUROSCI.21-14-05099.2001 PMC6762866

[B17] Hetts SW Rosen KM Dikkes P Villa-Komaroff L Mozell RL Expression and imprinting of the insulin-like growth factor II gene in neonatal mouse cerebellum J Neurosci Res 1997 50 6 958 966 10.1002/(SICI)1097-4547(19971215)50:6<958::AID-JNR6>3.0.CO;2-C 9452010

[B18] Fernandez C Tatard VM Bertrand N Dahmane N Differential modulation of Sonic-hedgehog-induced cerebellar granule cell precursor proliferation by the IGF signaling network Dev Neurosci 2010 32 1 59 70 10.1159/000274458 20389077 PMC2866582

[B19] Heijmans BT Kremer D Tobi EW Boomsma DI Slagboom PE Heritable rather than age-related environmental and stochastic factors dominate variation in DNA methylation of the human IGF2/H19 locus Hum Mol Genet 2007 16 5 547 554 10.1093/hmg/ddm010 17339271

[B20] Chowen JA Goya L Ramos S Busiguina S Garcia-Segura LM Argente J Pascual-Leone AM Effects of early undernutrition on the brain insulin-like growth factor-I system J Neuroendocrinol 2002 14 2 163 169 10.1046/j.0007-1331.2001.00758.x 11849376

[B21] Mares V Lodin Z Srajer J The cellular kinetics of the developing mouse cerebellum. I. The generation cycle, growth fraction and rate of proliferation of the external granular layer Brain Res 1970 23 3 323 342 10.1016/0006-8993(70)90060-0 5478301

[B22] Tiemeier H Lenroot RK Greenstein DK Tran L Pierson R Giedd JN Cerebellum development during childhood and adolescence: a longitudinal morphometric MRI study Neuroimage 2010 49 1 63 70 10.1016/j.neuroimage.2009.08.016 19683586 PMC2775156

[B23] Keller A Castellanos FX Vaituzis AC Jeffries NO Giedd JN Rapoport JL Progressive loss of cerebellar volume in childhood-onset schizophrenia Am J Psychiatry 2003 160 1 128 133 10.1176/appi.ajp.160.1.128 12505811

[B24] Kriaucionis S Heintz N The nuclear DNA base 5-hydroxymethylcytosine is present in Purkinje neurons and the brain Science 2009 324 5929 929 930 10.1126/science.1169786 19372393 PMC3263819

[B25] Kwan JS Kung AW Sham PC A simple bias correction in linear regression for quantitative trait association under two-tail extreme selection Behav Genet 41 5 776 779 21626281 10.1007/s10519-011-9475-0 PMC3162965

[B26] Fauque P Ripoche MA Tost J Journot L Gabory A Busato F Le Digarcher A Mondon F Gut I Jouannet P Modulation of imprinted gene network in placenta results in normal development of in vitro manipulated mouse embryos Hum Mol Genet 19 9 1779 1790 20150233 10.1093/hmg/ddq059

[B27] Smith RG Reichenberg A Kember RL Buxbaum JD Schalkwyk LC Fernandes C Mill J Advanced paternal age is associated with altered DNA methylation at brain-expressed imprinted loci in inbred mice: implications for neuropsychiatric disease Mol Psychiatry 2012 [Epub ahead of print] 10.1038/mp.2012.88 22733127

[B28] Didion JP Yang H Sheppard K Fu CP McMillan L de Villena FP Churchill GA Discovery of novel variants in genotyping arrays improves genotype retention and reduces ascertainment bias BMC Genomics 2012 13 34 10.1186/1471-2164-13-34 22260749 PMC3305361

