# Supplementary material for: DNA methylation at the Igf2/H19 imprinting control region is associated with cerebellum mass in outbred mice
Source: Mol Brain. 2012 Dec 6;5:42. doi: 10.1186/1756-6606-5-42 (PMC3541153; doi:10.1186/1756-6606-5-42)
Supplement: Additional file 1 — Supplementary Materials 1. Genomic sequences of each amplicon used in this study. Figure S1. No differences in DNA methylation at four additional imprinted loci (Kcnq1ot1, Mcts2, Nap1l5 and Sgce). Figure S2. High linkage disequilibrium (LD) across the SNPs genotyped in the murine Igf2/H19 region. Table S1. Details for the Sequenom EpiTYPER assays used to profile DNA methylation across DMRs associated with Kcnq1ot1, Mcts2, Nap1l5 and Sgce.Table S2. Stringent quality control and filtering steps used in analysis of Igf2/H19 DNA methylation data. Table S3. LD between the SNPs genotyped in Igf2 and H19. [file 1756-6606-5-42-S1.docx]

**Supplementary Materials 1** Genomic sequences of each amplicon used in this study. Annealing sites for primers are indicated by yellow and blue highlighting (forward and reverse respectively). CpG sites in the region are highlighted in red, and numbered according to the order in which they were assayed using the pyrosequencer. The sequences underlined in bold are the CTCF binding sites.

**CTCF1** chr7:149,767,865-149,768,017

AGGAGGTATAAGAATTTTGTAAGGAGATTATGTTTTATTTTTGGACGTTTGTT**GAATTAGTTGTGGGG**

**1 2 3 4 5 6**

**TTTATA**CGCGGGAGTTGTCGCGTGGTGGTAGTAAAATCGATTGCGTTAAATTTAAAGAGTTTTTTTAT

TTTTGGTATTGGAATTTATAAATGGTAATGTTGTGGGTTATTTAAGTTTAGTATTTT

**CTCF2** chr7:149,767,573-149,767,748

**6 5 4 3 2**

AAAGAATTTTTTGTGTGTAAAGATTAGGGTTGTCGTACGGCGGTAGTGAAGTTTCGTATATCGTAGTT

**1**

TTAAAC**GGATTGTAATTGATTGAGTT**TTTTTTTTTATTATTATTTATGATTTTATAGTTATGGGTTTT

ATGAGGTTAGGGGTTTATGTTAGTTTTTGAT

**CTCF3** chr7:149,766,533-149,766,776

GGGTTTTTTTGGTTATTGAATTTTAAAATTAGTTAGTGTGGTTTATTATAGGAAGGTATAGAAGT**TGT**

**1 2 3 4 5**

**TATGTGTAATAAGGGAA**CGGATGTTATCGCGCGGTGGTAGTATATTTTTATATATCGTGGTTTAAATG

**6**

TTGTTAATTTGGGGGGAGCGATTTATTTTTAGTAATATTTTAGGGTTATTTAAATAGGGATTTATAGG

GGTGGTAAGAGTGTGTATT

**CTCF4** chr7:149,766,109-149,766,322

**CTCF4_1 3 2**

GGGGTGGTATAATATATATTTTTTGGGTAGTTTTTTTAGTTTTGCGTTTTTTACGATCGATCGGTTTA

**1**

TTTTTTACG**TTGTGTAGATTTGGTTATAG**TTAAATGGATAGACGATGTCGCGTGGTGGTAGTATAATA

TTATATATTGTTCGGTAGACGCGGTATAGGTTGGGGTTC**GTTGTGATAAAGTTTTGAGT**ATTTTAGGT

TTAATAAAGGGATTAGGTATTTGTGTATTTACGGAATGGTTTTTTTTTGT

**CTCF4_2**

GGGGTGGTATAATATATATTTTTTGGGTAGTTTTTTTAGTTTTGCGTTTTTTACGATCGATCGGTTTA

**7 6 5**

TTTTTTACG**TTGTGTAGATTTGGTTATAG**TTAAATGGATAGACGATGTCGCGTGGTGGTAGTATAATA

**4 3 2 1**

TTATATATTGTTCGGTAGACGCGGTATAGGTTGGGGTTC**GTTGTGATAAAGTTTTGAGT**ATTTTAGGT

TTAATAAAGGGATTAGGTATTTGTGTATTTACGGAATGGTTTTTTTTTGT

**Supplementary Figure 1** No differences in DNA methylation were observed across DMRs associated with four additional imprinted loci (*Kcnq1ot1*, *Mcts2*, *Nap1l5* and *Sgce*) indicating the observed effect is specific to the regulation of *Igf2* and *H19.*

**
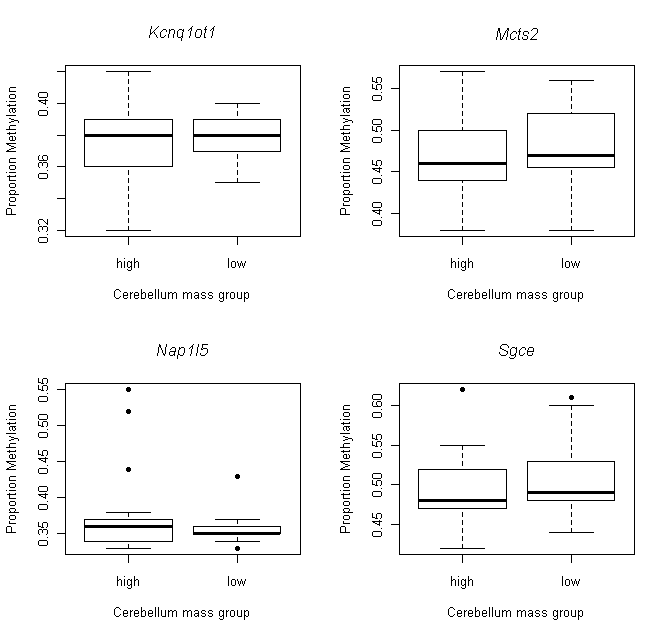
**

p=0.27

p=0.28

p=0.38

p=0.60

**Supplementary Figure 2** High linkage disequilibrium (LD) across the SNPs genotyped in the murine *Igf2/H19* region.

**
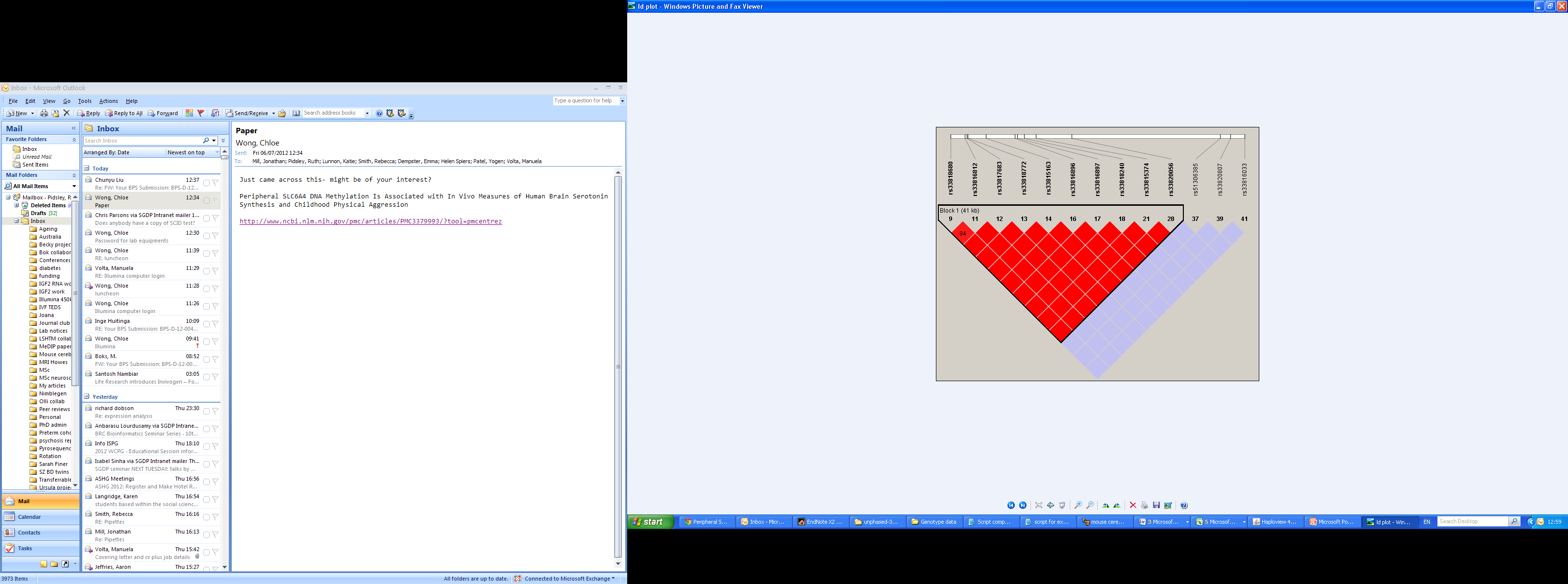
**

**Supplementary Table 1** Details for the Sequenom EpiTYPER assays used to profile DNA methylation across DMRs associated with *Kcnq1ot1*, *Mcts2*, *Nap1l5* and *Sgce*

| **Assay Name** | **Genomic Position** | **Left Primer*** | **Right Primer**** | **PCR** | **Fragments** |
| --- | --- | --- | --- | --- | --- |
|  | **(mm9)** |  |  | **Length (bps)** | **(CpGs Covered)** |
| Kcnq1ot1 | chr7:150481246-150481473 | GTATTTTATTTATTATTTTGGTGTTGGTT | CCCCATCTTTATAACCCAAACTTAC | 227 | 10 (11) |
| Mcts2 | chr2:152512486-152512694 | GAGTTGAGGGATGGATAGTTATTT | CAAAATTCCCAAACATTAAAATACTAAAA | 208 | 15 (17) |
| Nap1l5 | chr6:58856789-58857051 | TTATAAAGTTTTTTTTGGGTTTGGG | CTACAAAACCTCTCTAAACCAACTCT | 262 | 9 (23) |
| Sgce | chr6:4697698-4697923 | AAATTTTGTTAAGTTTTTAGTGGTTAGA | CTTAAAAATACAAAACCAATCACTTT | 225 | 11 (16) |

**Supplementary Table 2** Stringent quality control and filtering steps used in analysis of *Igf2/H19* DNA methylation data. DNA samples with less than 60% complete measurement success rate per amplicon were discarded. CpG fragments potentially confounded by a SNP or CpG fragments with a measurement success rate below 80% were discarded.

| Amplicon | Excluded samples (n) | Excluded CpG sites |
| --- | --- | --- |
| CTCF1 | 7 |  |
| CTCF2 | 4 | CpG 3 |
| CTCF 3 | 9 | CpG 6 |
| CTCF4_1 | 3 |  |
| CTCF4_2 | 1 |  |

**Supplementary Table 3** LD between the SNPs genotyped in *Igf2* and *H19*

|  | **rs33818680** | **rs33816812** | **rs33817683** | **rs33818772** | **rs33815163** | **rs33816896** | **rs33816897** | **rs33818240** | **rs33815374** | **rs33820056** | **rs51306385** | **rs33820807** | **rs33816033** |
| --- | --- | --- | --- | --- | --- | --- | --- | --- | --- | --- | --- | --- | --- |
| **rs33818680** |  | 0.883 | 0.942 | 0.942 | 0.942 | 0.942 | 0.116 | 0.942 | 0.942 | 0.778 | 0.03 | 0.005 | 0.005 |
| **rs33816812** | 0.940 |  | 0.942 | 0.942 | 0.942 | 0.942 | 0.116 | 0.942 | 0.942 | 0.778 | 0.03 | 0.005 | 0.03 |
| **rs33817683** | 1 | 1 |  | 1 | 1 | 1 | 0.109 | 1 | 1 | 0.826 | 0.031 | 0.005 | 0.005 |
| **rs33818772** | 1 | 1 | 1 |  | 1 | 1 | 0.109 | 1 | 1 | 0.826 | 0.031 | 0.005 | 0.005 |
| **rs33815163** | 1 | 1 | 1 | 1 |  | 1 | 0.109 | 1 | 1 | 0.826 | 0.031 | 0.005 | 0.005 |
| **rs33816896** | 1 | 1 | 1 | 1 | 1 |  | 0.109 | 1 | 1 | 0.826 | 0.031 | 0.005 | 0.005 |
| **rs33816897** | 1 | 1 | 1 | 1 | 1 | 1 |  | 0.109 | 0.109 | 0.09 | 0.003 | 0.047 | 0.003 |
| **rs33818240** | 1 | 1 | 1 | 1 | 1 | 1 | 1 |  | 1 | 0.826 | 0.031 | 0.005 | 0.005 |
| **rs33815374** | 1 | 1 | 1 | 1 | 1 | 1 | 1 | 1 |  | 0.826 | 0.031 | 0.005 | 0.005 |
| **rs33820056** | 1 | 1 | 1 | 1 | 1 | 1 | 1 | 1 | 1 |  | 0.038 | 0.004 | 0.004 |
| **rs51306385** | 1 | 1 | 1 | 1 | 1 | 1 | 1 | 1 | 1 | 1 |  | 0 | 0 |
| **rs33820807** | 1 | 1 | 1 | 1 | 1 | 1 | 1 | 1 | 1 | 1 | 1 |  | 0 |
| **rs33816033** | 1 | 1 | 1 | 1 | 1 | 1 | 1 | 1 | 1 | 1 | 1 | 1 |  |
| **MAF** | 0.300 | 0.300 | 0.288 | 0.288 | 0.288 | 0.288 | 0.212 | 0.288 | 0.288 | 0.250 | 0.012 | 0.012 | 0.012 |
| **HWE p-val** | 1 | 1 | 1 | 1 | 1 | 1 | 0.2292 | 1 | 1 | 0.0818 | 1 | 1 | 1 |
